# Clinical Profile and Prognosis of Hereditary Transthyretin Amyloid Cardiomyopathy: A Single-Center Study in South China

**DOI:** 10.3389/fcvm.2022.900313

**Published:** 2022-06-27

**Authors:** Shuai Wang, Wenke Peng, Min Pang, Ling Mao, Daoquan Peng, Bilian Yu, Sha Wu, Die Hu, Yang Yang, Jia He, Mingqi Ouyang

**Affiliations:** Department of Cardiovascular Medicine, Second Xiangya Hospital, Central South University, Changsha, China

**Keywords:** hereditary, transthyretin amyloidosis, cardiomyopathy, prognosis, China

## Abstract

**Background:**

Hereditary transthyretin amyloid cardiomyopathy (ATTR-CM) is a genotypically heterogeneous disorder with a poor prognosis. There is limited literature describing the variants responsible for ATTRv in areas outside the United State, the United Kingdom and Europe. This study was performed to describe the clinical characteristics and genotypic profiles of this disease in South China.

**Methods:**

This was a single-center retrospective study that evaluated 29 patients with a confirmed diagnosis of hereditary transthyretin amyloid cardiomyopathy enrolled from January 2016 to November 2021.

**Results:**

93.1% patients were male and the median age of symptom onset was 53 (46, 62.5) years old. The initial manifestations of ATTR-CM were cardiovascular symptoms (55.2%), neuropathy (41.4%) and vitreous opacity (3.4%). Phenotypes at diagnosis were mixed (82.8%), predominant cardiac (6.9%), neurological (6.9%) and ophthalmic (3.4%). Poor R-wave progression (41%), pseudo-infarct (31%) and low-voltage (31%) patterns were common findings on electrocardiogram. Unexplained increased wall thickness was observed in all 29 patients, with mean septal and posterior wall thicknesses of 14.25 ± 6.26 mm and 15.34 ± 2.84 mm, respectively. Diastolic dysfunction was also seen in all 29 patients, and 17 (58%) had a restrictive fill pattern at diagnosis. Nine different missense mutations of the TTR gene were found in 29 patients from 23 families, with c.349G>T (p.Ala117Ser) the most common mutation. The median survival time after diagnosis was 47.6 (95% CI 37.9-57.4) months, with 1, 3 and 5-year survival rates of 91.2%, 74% and 38% respectively. Patients with advanced heart failure (National Amyloidosis Staging stage II/III) had worse survival than stage I [Breslow (Generalized Wilcoxon), χ2 = 4.693, *P* = 0.03)].

**Conclusions:**

ATTR amyloidosis genotypes and phenotypes are highly heterogeneous. Advanced heart failure predicts a poor prognosis. Understanding the different clinical profiles of ATTR cardiac amyloidosis with different genotype is important to its early recognition.

## Introduction

Hereditary transthyretin amyloidosis (ATTRv) represents a group of severe diseases with a broad spectrum of genotypes and phenotypes caused by transthyretin (TTR) gene mutations. ATTR is the result of dissociation of the transthyretin tetramer into monomers that mis-fold, forming amyloid deposits in the extracellular space (1). The most common manifestations of ATTR are polyneuropathy and cardiomyopathy (2). As our understanding of the molecular mechanisms behind ATTR amyloidosis improves, disease-modifying treatments including stabilizing molecular (tafamidis) and genetic silencers (partisiran and inotersen) have been made available. As pharmacotherapy is more effective in the early stages of the disease, the early diagnosis of ATTR by understanding its genotype-phenotype correlations is important (3–5).

More than 120 mutations of the TTR gene have been described, and as of December 2021 there are 68 pathogenic/likely pathogenic variants of the TTR gene listed in the National Center for Biotechnology Information ClinVar database (6–8). Some genotype-phenotype variability has been reported. Although the THAOS registry collected ATTR phenotypes and genotypes from continental Western Europe and the United States, few studies have been performed in China. Two recent retrospective cohort studies revealed that the mutation of TTR in Chinese patients may be quite different from that seen in the United States and Europe (9, 10). Further, those two studies mainly discussed ATTRv patients from northern China and one study focused on ATTRv patients with neuropathy as main symptom. Here, we report the phenotype and genotype of 29 patients with ATTRv cardiomyopathy (ATTRv-CM) from 23 unrelated South Chinese families.

## Methods

### Study Design and Population

This was a descriptive, observational, and retrospective study that included patients diagnosed with hereditary ATTRv-CM at our institution from January 2016 to November 2021. The study was undertaken in accordance with the Declaration of Helsinki and was approved by the ethics committee of Second Xiangya Hospital. Due to the retrospective nature of this study, informed consent was waived for patients who had died. Otherwise, all patients provided written informed consent.

### Diagnosis of Hereditary ATTR Cardiac Amyloidosis

The diagnosis of hereditary ATTRv-CM was established by clinical presentation, family history, echocardiography, tissue biopsy and DNA sequencing for the presence of a mutation in the TTR gene. Cardiac magnetic resonance (CMR) and ^99m^technetium (^99m^Tc)-pyrophosphate (PYP) scintigraphy were performed in select cases. All included patients had a positive tissue biopsy (endocardium or extracardiac, e.g., abdominal adipose tissue or vitreous body) for amyloidosis and a pathogenic/likely pathogenic TTR variant. Light chain amyloidosis was ruled out by confirming a normal free light chain ratio and the absence of detectable serum and/or urine monoclonal protein. Patients without a related gene mutation on TTR gene sequencing were excluded.

The date of symptom onset was reported by the patient as the date on which symptoms related to amyloidosis first were noted. The date of diagnosis was the date on which the diagnosis of amyloidosis was confirmed histologically.

Phenotype categories based on clinical presentation at the time of diagnosis were: 1) predominantly cardiac. 2) predominantly neurological. 3) mixed (cardiac and neurological); and 4) ophthalmic. Patients with a predominantly cardiac phenotype were those (1) with heart failure, dyspnea and/or abnormal electrocardiogram findings caused by rhythm disturbance and (2) who did not have more than mild neurological or gastrointestinal symptoms (excluding erectile dysfunction, constipation and carpal tunnel syndrome). Patients with a predominantly neurologic phenotype were those with (1) walking disability of any severity, other neurologic symptoms of any severity or gastrointestinal symptoms (early satiety, nausea, vomiting, unintentional weight loss, diarrhea, constipation or fecal incontinence) of any severity and (2) who did not have heart failure, dyspnea, or an abnormal ECG due to a rhythm disturbance. Patients with a (3) mixed phenotype were patients who had at least 1 cardiac and neurological symptom as described above. Patients with (4) an ophthalmic phenotype had vitreous opacity with none of the cardiac or neurological symptoms described above.

Cardiomyopathy was defined as an end-diastolic thickness of the left ventricular wall > 1.2 cm (in the absence of any other plausible causes of LV hypertrophy). Other clues suggestive of cardiac amyloidosis included granular sparkling appearance of the ventricular myocardium, increased thickness of the atrioventricular valves or the interatrial septum and reduced tissue doppler e' velocities. CMR and ^99m^Tc-PYP were performed in select cases. Characteristic CMR findings of (1) the inability to suppress or “null” the myocardial signal or (2) the presence of diffuse subendocardial or transmural enhancement patterns on late gadolinium enhancement CMR were recorded. Grade 2 or 3 cardiac uptake on ^99m^Tc-PYP scintigraphy was also recorded.

N-terminal pro-B type natriuretic peptide (NT-proBNP), estimated glomerular filtration rate (eGFR), cardiac troponin (cTnT), modified poly neuropathy disability (PND) score and the U.K. National Amyloidosis Staging (NAC) and New York Heart Association (NYHA) stages were recorded. NAC Stage I is defined as NT-proBNP ≤ 3000 ng/L and eGFR ≥ 45 mL/min, and NAC stage III was defined as NT-proBNP> 3000 ng/L and eGFR < 45 mL/min. The remainder of the patients were assigned to NAC stage II.

### Neurological Work-Up

Neurological examination included an assessment of motor, cerebellar and reflex function as well as screening for sensory function. Muscle strength was documented according to the Medical Research Council (MRC) scale (5/5 normal strength, 0/5 no contractions). Motor and sensory nerve conduction studies were performed on patients with reported neurologic symptoms. The PND score was used to stage patients with ATTRv and polyneuropathy at baseline. PND I was defined as sensory disturbances in the extremities but preserved walking capacity; PND II was defined as difficulty walking but without the need for a walking stick; PND III was defined as sticks or crutches required for walking; and PND IV was defined as confined to a wheelchair or bed.

### Genetic Testing of the TTR Gene

Genomic DNA was isolated from whole peripheral blood using standard techniques. Exon 1–4 of the *TTR* gene were amplified with polymerase chain reaction. Sequences of the *TTR* gene (NM_000371.3; NG_009490.1) were analyzed using the Applied BioSystems SeqScape software v4.0 (Carlsbad, USA) and the DNA Dynamo Sequence Analysis Software (North Wales, UK). The datasets presented in this study can be found in an online repository. The name of the repository and its accession numbers can be found in the supplementary material (Supplementary Table 1). Genetic testing and clinical penetrance assessments were performed on family members for probands if available.

### Follow-Up

Patient follow-ups at 6 to 12-month intervals included an ECG, NT-proBNP and yearly echocardiogram. Mutation carriers were surveilled via regular (yearly) telephone contact.

### Statistical Analyses

Continuous variables are reported as mean ± SD or median and interquartile range (IQR) (for non-normal distributions). Categorical variables are reported as percentages. Kaplan-Meier survival was calculated from the date of the original diagnosis to the date of death or the most recent contact. Gehan-Breslow-Wilcoxon method was used to compare survival curves. All tests were 2-tailed and a *P*-value <0.05 was considered statistically significant. Statistical analyses were performed using SPSS Statistics (version 26. IBM Corp., Chicago, IL, USA).

## Results

### Clinical Characteristics of Patients With ATTR Cardiac Amyloidosis

The clinical characteristics at diagnosis of the 29 patients are summarized in Tables 1, 2. Twenty-seven (93.1%) patients were male. The median age at symptom onset was 53 (46, 62.5) years and the median age at diagnosis was 56 (47.75–66.25) years. The median time from symptom onset to diagnosis was 24 (12−24) months and the median course from diagnosis to the last visit was 15 (6.5–37.5) months.

**Table 1:** Clinical Manifestations of ATTRv-CM and the *TTR* variant Ala97Ser.

	**All (*n* = 29)**	**Ala97Ser (*n* = 11)**	**Non Ala97Ser (*n* = 18)**	***p*-value**
**General characteristics**				
Male, *n* (%)	27 (93.1)	10 (91)	17 (89.5)	0.702
Age of symptom onset, y	53 (46–62.5)	65 (62–66)	50 (45–58.5)	<0.001
Age at diagnosis, y	56 (47.8–66.3)	68 (64–72.5)	50.5 (46.8–59.3)	<0.001
Time from symptom onset to diagnosis, m	24 (12–48)	48 (24–102)	24 (13–45)	0.084
Course from diagnosis to last visit, m	15 (6.5–37.5)	13 (8–30)	19 (5.75–45.75)	0.65
**Initial presentation**				
Neuropathy as initial presentation, *n* (%)	12 (41.4)	11 (100)	15 (83.3)	0.27
Cardiovascular symptom as initial presentation, *n* (%)	16 (55.2)	0 (0)	16 (88.9)	<0.001
Vitreous opacity, *n* (%)	1 (3.4)	0 (0)	1 (5.6)	1.0
**Clinical presentation at diagnosis**				
HF, *n* (%)	23 (79.3)	8 (72.7)	15 (88.2)	0.35
NYHA stage, *n* (%)				
I	7 (24.1)	3 (27)	4 (36.4)	0.65
II	1 (3.4)	1 (9.1)	0 (0)	0.38
III	14 (48.3)	5 (45.5)	9 (52.9)	1.0
IV	7 (24.1)	2 (18.2)	5 (29.4)	0.68
Typical echocardiography at diagnosis, *n* (%)	29 (100)	11 (100)	19 (100)	1.0
Abnormal ECG, *n* (%)	15 (51.7)	5 (45.5)	10 (55.6)	0.71
NAC staging score, *n* (%)				
Stage I	21 (67.7)	8 (72.7)	12 (70.6)	1.0
Stage II	6 (19.4)	3 (27.3)	3 (17.6)	0.65
Stage III	4 (12.9)	0 (0)	2 (11.8)	0.51
Somatic neuropathy, *n* (%)				
(Diarrhea, Constipation, Orthostatic hypotension, Upper	14 (48.3)	6 (54.5)	8 (47.1)	0.70
gastrointestinal tract symptoms, eg. early satiety, dyspepsia,				
dysphagia, vomiting)				
Peripheral neuropathy, *n* (%)	25 (86.2)	11 (100)	14 (82.4)	0.26
History of Carpal tunnel syndrome, *n* (%)	6 (20.7)	2 (18.2)	4 (23.5)	0.73
Lumber Spinal stenosis, *n* (%)	1 (3.4)	1 (9.1)	0 (0)	0.38
PND score at diagnosis				
0	4 (12.9)	0 (0)	4 (22.2)	0.27
I	8 (25.8)	1 (9.1)	7 (38.9)	0.11
II	9 (29)	3 (27.3)	6 (35.3)	0.001
III	7 (22.6)	6 (54.5)	1 (5.9)	0.006
IV	3 (9.7)	1 (9.1)	2 (11.8)	0.14
**Phenotype**				
Predominantly cardiac, *n* (%)	2 (6.9)	0 (0)	2 (11.1)	0.51
Predominantly neurological, *n* (%)	2 (6.9)	1 (9)	1 (5.6)	1.0
Mixed, *n* (%)	24 (82.8)	10 (91)	14 (77.8)	0.62
Ocular, *n* (%)	1 (3.4)	0 (0)	1 (5.6)	1.0
Patients receiving tafamidis, *n* (%)	4 (9.5)	3 (25%)	1 (5.6)	0.14

*Values are mean ± SD, n (%), or median (25th to 75th percentile). HF, heart failure; NYHA, New York Heart Association; NAC, National Amyloidosis Staging; PND, polyneuropathy disability*.

**Table 2:** Complementary baseline tests for ATTRv–CM and the *TTR* variant Aal97Ser.

	**All (*n* = 29)**	**Aal97Ser (*n* = 11)**	**Non–Ala97Ser (*n* = 18)**	***p*–value**
**Biomarkers**				
NT–proBNP, pg/ml	2789 (1206–5024)	2081 (973–3706.3)	2722 (1425–5245)	0.30
cTnT, pg/ml	61.5 (42.8–88.9)	45.5 (42.1–73.7)	85.3 (27.7–125.5)	0.41
eGFR, ml/min/1.73 m^2^	81.7 (66.4–93.6)	90 (83.7–95.4)	79.3 (64.5–94.6)	0.16
**Electrocardiogram**				
Atrial fibrillation/flutter, *n* (%)	2 (7)	0 (0)	2 (25)	0.51
AV block, *n* (%)	5 (17)	0 (0)	5 (27.8)	0.13
RBBB/LBBB, *n* (%)	3 (10)	1 (9.1)	2 (25)	1.0
Pacemaker, *n* (%)	2 (7)	0 (0)	2 (25)	0.51
LV hypertrophy (Sokolow), *n* (%)	0 (0)	0 (0)	0 (0)	1.0
Low voltage				
Classic criteria^a^, *n* (%)	9 (31)	5 (45.5)	4 (22)	0.24
Sokolow^b^, *n* (%)	8 (58)	3 (27.3)	5 (27.8)	1.0
Pseudo–infarct pattern, *n* (%)	9 (31)	3 (27.3)	6 (33.3))	1.0
Poor Precordial R wave progression, *n* (%)	12 (41)	5 (45.5)	7 (38.9)	1.0
**Echocardiography**				
LVH, *n* (%)	29 (100)	11 (100)	18 (100)	1.0
LVEF <50%, *n* (%)	2 (6.8)	1 (9.1)	1 (5.6)	1.0
LV diastolic dysfunction, *n* (%)	29 (100)	11 (100)	18 (100)	1.0
Restrictive filling (e' <5 cm/s)	17 (58)	6 (54.5)	11 (61.1)	1.0
Pericardial effusion, *n* (%)	9 (31)	3 (27.3)	6 (33.3)	
LVIDd, mm	47.74 ± 5.37	43.16 ± 3.18	43.33 ± 4.50	0.99
IVS thickness, mm	16.39 ± 3.04	15.83 ± 4.26	17.00 ± 2.60	0.59
PWT, mm	15.34 ± 2.84	14.50 ± 2.26	17.00 ± 2.61	0.08
Left atrial size, mm	39.47 ± 5.54	38.83 ± 5.51	43.50 ± 4.46	0.68
LVEF, %	52.65 ± 10.65	55.17 ± 13.87	49.83 ± 5.12	0.70
PA systolic pressure, mm Hg	33.36(26.16–35.18)	32.00 (26.16–34.59)	28.00 (24.36–30.00)	0.37
e' average, cm/s	4.64 ± 1.11	5.33 ± 1.25	4.07 ± 0.62	0.05
**Cardiac magnetic resonance at diagnosis**	***n*** **=** **17**	***n*** **=** **5**	***n*** **=** **12**	
Inability to suppress or “null” the myocardial signal, *n* (%)	17 (100)	5 (100)	12 (100)	1.0
Diffuse subendocardial/transmural enhancement pattern, *n* (%)	17 (100)	5 (100)	12 (100)	1.0
**Cardiac uptake grading in** ^**99m**^**Tc–PYP scintigraphy at diagnosis**	***n*** **=** **9**	***n*** **=** **5**	***n*** **=** **4**	
Grade 0	0 (0)	0 (0)	0 (0)	
Grade 1	0 (0)	0 (0)	0 (0)	
Grade 2	2 (22.2)	1 (20)	1 (25)	1.0
Grade 3	7 (77.8)	4 (80)	3 (75)	1.0

*Values are mean ± SD, n (%), or median (25th to 75th percentile). NT–proBNP, N–terminal pro–B type natriuretic peptide; cTnT, cardiac troponin; eGFR, estimated glomerular filtration rate; AV, atrioventricular; RBBB, right bundle branch block; LBBB: left bundle branch block; LV, left ventricular; LVH, left ventricular hypertrophy; LVEF, left ventricular ejection fraction; LVIDd, left ventricular internal diameter at end–diastole; IVS, interventricular septum; PWT, posterior wall thickness; PA, pulmonary artery*.

*^a^ <0.5 mV in all limb leads or < 1.0 mV in all precordial leads*.

*^b.^sum of S in V1 and R in V5/V6 < 25mm*.

Dyspnea on exertion and limited physical capacity were the initial complaints that prompted a comprehensive cardiological examination in 16 cases (55.2%). Another 12 (41.4%) patients presented with distal sensory disturbances and/or weakness predominantly of the lower limbs. One patient reported vitreous opacity as their initial manifestation and had asymptomatic cardiac hypertrophy at diagnosis.

Twenty-four (82.8%) patients presented with a mixed phenotype that included both cardiomyopathy and neuropathy, while 2 (6.9%) had a predominantly cardiac phenotype and 2 (6.9%) had a predominantly neurological phenotype. One patient (3.4%) presented with vitreous opacity with asymptomatic myocardial hypertrophy. Peripheral and autonomic nerve dysfunction were found in 25 (86.2%) and 14 (48.3%) subjects at diagnosis, respectively. Six patients (20.7%) had a history of uni- or bilateral carpal tunnel syndrome. Lumber spinal stenosis was reported in one patient. The PND score was used to describe the severity of the peripheral neuropathy (Table 1). At diagnosis, 72.4% (21) had severe heart failure (NYHA III/IV), 3.4% (1) had mild heart failure (NYHA II) and 24.1% (7) had asymptomatic myocardial hypertrophy (Table 1). Elevated NT-proBNP and cTnT were found in 29 (100%) and 28 (97%) patients, respectively. The median NT-proBNP and cTnT levels were 2789 (1206–5024) pg/ml and 61.5 (42.8–88.9) pg/ml at diagnosis. Only one patient had an eGFR <45 and the median eGFR was 81.7 (66.4–93.6) ml/min/1.73 m^2^ (Table 2). Of the 29 patients, 19 (65.6%) were NAC stage I, 9 (31%) stage II and 1 (3.4%) stage III.

Electrocardiogram abnormalities were evident in 15 (51.7%) patients at diagnosis. Two (6.9%) had atrial fibrillation, 5 (17.2%) presented with an atrial-ventricular block and 2 went on to require a permanent pacemaker. The classic low-voltage pattern of ATTR-CM was found in 9 (31%) patients, while a low-voltage pattern according to the Sokolow criteria was found in 8 (27.5%). A pseudo-infarct pattern was found in 9 (31%) patients and poor R wave progression was found in 12 (41%, Table 2).

Unexplained increased wall thickness (>12 mm) and diastolic dysfunction were observed in 29 (100%) patients. Two (6.9%) had systolic dysfunction with an ejection fraction <50%. Restrictive filling (e' <5 cm/s) was present in 17 (58%) patients at diagnosis. Pericardial effusions were present in 9 patients (31%). The mean septal and posterior wall thicknesses of the cohort were 16.39±3.04 mm and 15.34±2.84 mm, respectively, and the mean systolic left atrial diameter was 39.47±5.54 mm. The mean LVEF was 52.65±10.6%. CMR was performed on 16 (55.2%) patients to diagnose ATTR cardiac amyloidosis, all of which showed diffuse subendocardial or transmural LGE and abnormal gadolinium kinetics. ^99m^TC-PYP scintigraphy was performed on 9 patients (31%). Seven had grade 3 cardiac uptake and 2 patients had grade 2 uptake. Biopsies were performed on all patients, and originated from the abdominal fat (28), endomyocardium (1) or vitreous body (1) (Table 2). All samples were positive on Congo red staining and TTR immunochemistry. Four patients (9.5%) in this cohort were on tafamidis post-enrollment, and no patient received a liver transplant.

### Genetic Spectrum of TTR Mutations

**Figure 1:**
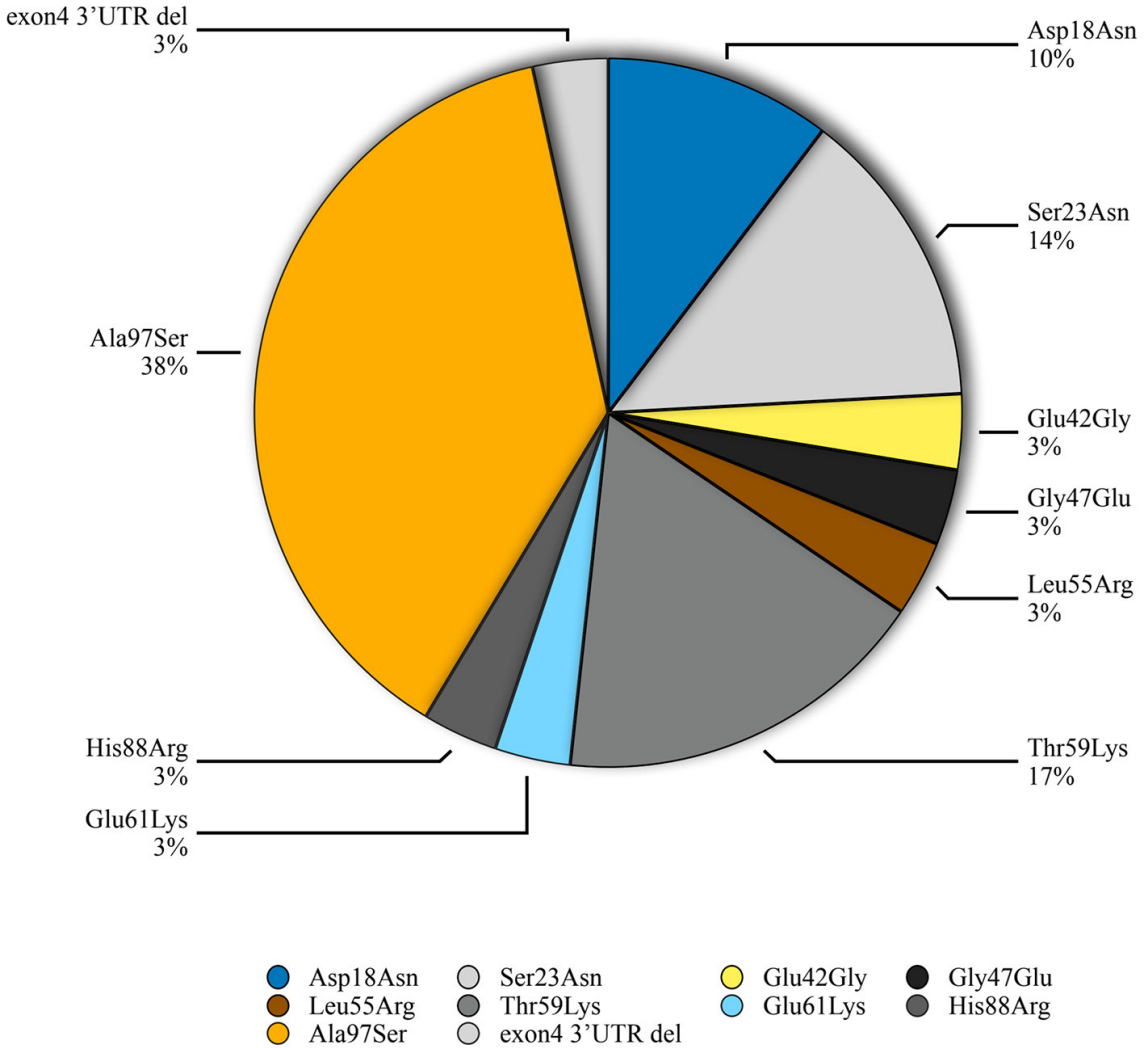
Distribution of *TTR* mutations in a cohort of ATTR-CA patients from South China.

Genetic testing of the probands and their family members identified 9 different missense *TTR* mutations [Asp18Asn(p.Asp38Asn), Ser23Asn(p.Ser43Asn), Glu42Gly (p.Glu62Gly), Gly47Glu(p.Gly67Glu), Leu55Arg(p.Leu75Arg), Thr59Lys(p.Thr79Lys), Glu61Lys(p.Glu81Lys), His108Arg (p.His108Arg), Ala97Ser(p.Ala117Ser)] and 1 small deletion mutation (3'UTR c.624_632 del GACTTCTCC) in 29 patients from 23 families. Pedigree analysis found another 11 asymptomatic mutation carriers without electrocardiographic and echocardiographic abnormalities. The predominant genotypes identified in ATTR-CA were the Ala97Ser (p.Ala117Ser) mutation (11/29, 37.9%) followed by Thr59Lys (p.Thr79Lys) (5, 17.2%) (Figure 1). Of these variants, Asp18Asn, Ser23Asn, Glu42Gly, Gly47Glu, Thr59Lys, Glu61Lys, His88Arg and Ala97Ser were associated with the mixed phenotype while Leu55Arg was associated with the ophthalmic phenotype. The deletion mutation was found in a patient with a cardiac phenotype (Table 3).

**Table 3:** Genotype distribution in probands and family members.

***TTR* mutation**	**Overall *N = 40, n (%)***	**Symptomatic patients *N = 29, n (%)***	**Associated phenotype in the cohort**
A97S	16 (40)	11 (37.9)	N, H
D18N	4 (10)	3 (10.3)	N, H
E42G	1 (2.5)	1 (3.4)	N, H
E61K	1 (2.5)	1 (3.4)	N, H
G47E	1 (2.5)	1 (3.4)	N, H
H88R	1 (2.5)	1 (3.4)	N, H
L55R	1 (2.5)	1 (3.4)	E, H
S23N	4 (10)	1 (3.4)	N, H
T59K	8 (20)	5 (17.2)	N, H
Exon3 3'UTR c.624_632delGACTTCTCC	1 (2.5)	1 (3.4)	H

*N, neurological; H, Heart; E, eye*.

Ala97Ser was the most common mutation in this cohort. 72.7% (8/11 cases) of patients carrying the Ala97Ser presented with evidence of heart failure at confirmed diagnosis of ATTRv cardiac amyloidosis, with 63.7% (7/11) having NYHAIII/IV (Table 1). All patients with Ala97Ser mutations initially presented with neurological dysfunction, but cardiac symptoms followed 2–9 years later. The median age of onset of neurological symptom related to ATTR amyloidosis in patients carrying the Ala97Ser variant was 65 (62–67) years old and the median time from neurological symptom onset to diagnosis of ATTRv-CM was 48 (24–102) months.

### Survival

**Figure 2:**
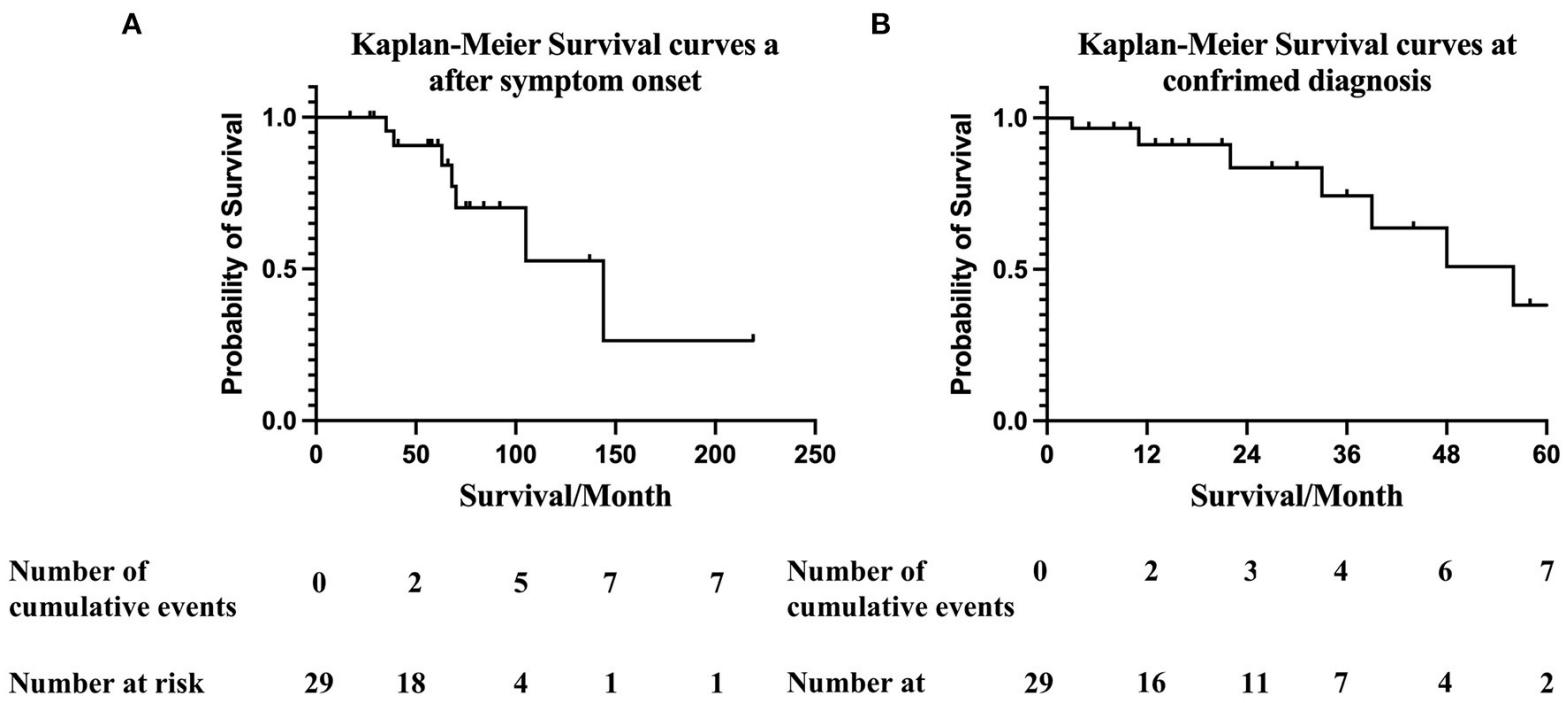
**(A)** Kaplan-Meier survival curves of data obtained from ATTR-CA symptom onset. Overall survival at 12-, 36-, 60-, and 120- months after symptom onset were 100%, 90%, 70% and 52.6%, respectively. Numbers below the plot represent the number of subjects at risk at each time point. Cumulative events refer to all-cause death. The median survival time after symptom onset was 131 (95%CI 88.6–173.7) months. **(B)** Kaplan-Meier survival curves at confirmed diagnosis. Overall survival at 12-, 36-, and 60- months was 91.2%, 74%, and 38% respectively. Numbers below the plot represent the number of subjects at risk at each time point. Cumulative events refer to all-cause death. The estimated median survival time after confirmed diagnosis of ATTRv-CM was 47.6 (95%CI 37.9–57.4) months.

During January 2016 to November 2021, 29 patients were diagnosed with hereditary ATTRv-CM and followed up at our institution. The date when symptoms related to ATTRv began, (which was 30 (12, 54) months before diagnosis of ATTR-CM), was recorded according to the patient's report. The mortality rate of the cohort over a median follow-up of 14 (5.75, 32.25) months was 7/29 (24%). The estimated median survival times after initial symptom onset related to ATTRv and at confirmed diagnosis of ATTRv-CM by Kaplan-Meier survival curve analysis were 131 (95%CI 88.6–173.7) and 47.6 (95%CI 37.9–57.4) months, respectively. According to Kaplan-Meier survival curve analysis, the estimated 3-, 5- and 10-year survival rates after symptom onset were 90%, 70% and 52.6%, respectively (Figure 2A). The overall 1-year survival rate after diagnosis was 91.2%, and the 3- and 5-year survival rates were 74% and 38%, respectively (Figure 2B).

**Figure 3:**
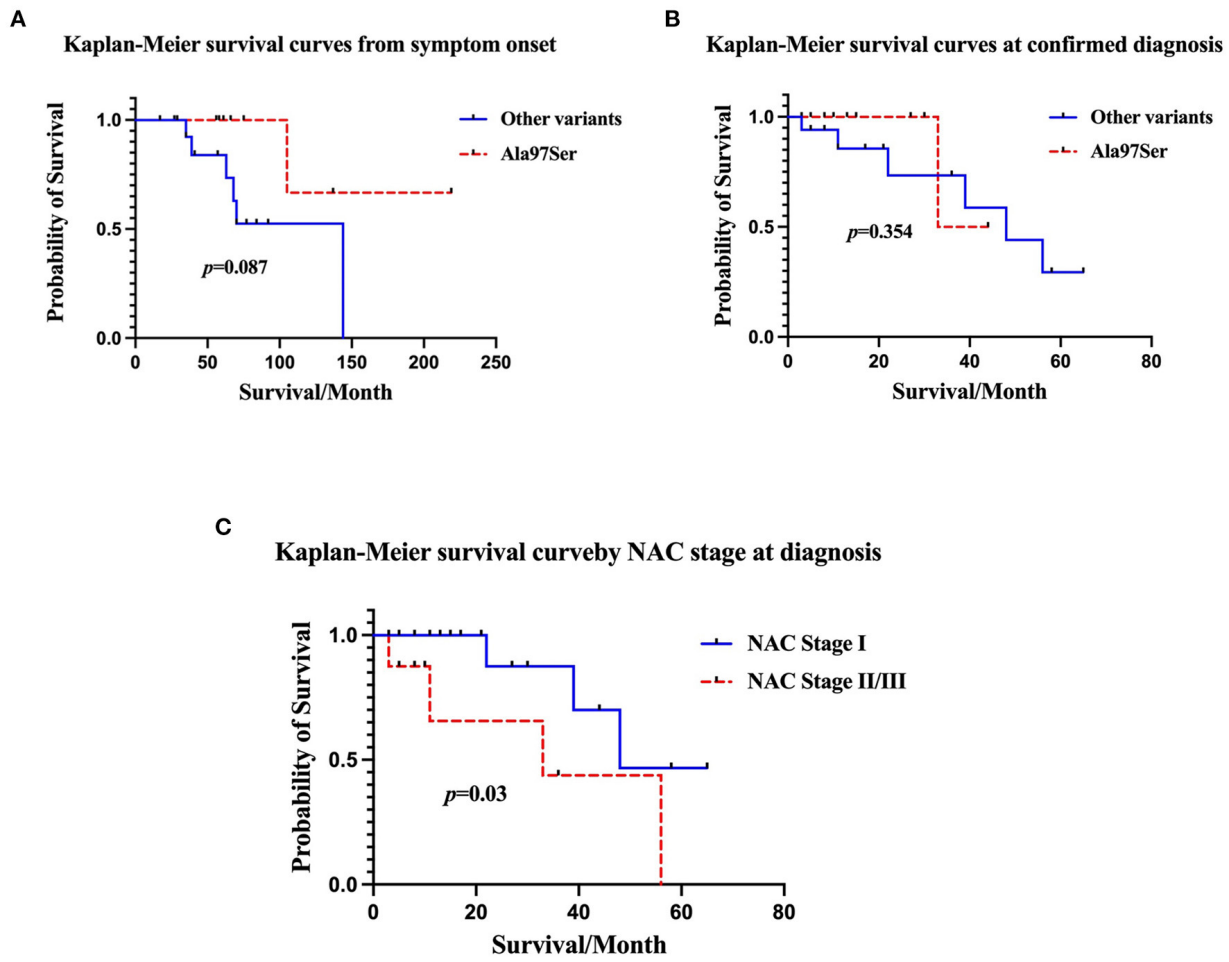
**(A)** Comparison of Kaplan-Meier survival curves of data obtained from ATTR-CA symptom onset between patients with Ala97Ser and other variants. There was no significant difference in survival in patients with Ala97Ser and other variants [Breslow (Generalized Wilcoxon), χ2 = 2.92, *p* = 0.087]. **(B)** Comparison of Kaplan-Meier survival curves at confirmed diagnosis between patients with Ala97Ser and other variants. Survival after diagnosis was not significantly different in patients with Ala97Ser and other variants [Breslow (Generalized Wilcoxon), χ2 = 0.859, *p* = 0.35]. **(C)** Kaplan-Meier survival curve for patients grouped by NAC stage at diagnosis. Patients with NAC stage II/III disease had worse survival than those with NAC stage I disease [Breslow (Generalized Wilcoxon), χ2 = 4.693, *p* = 0.03)]. The median survival time of patients in NAT stage I was 48 months (95%CI 39–63) and the cumulative survival at 60 months was 46.7%. The median survival time of patients in NAC stage II/III was 33 months (95%CI 13.7–55.3) and the cumulative survival at 60 months was 0%.

For patient carrying Ala97Ser variant, survival curve analysis revealed the estimated mean survival time after symptom onset and at diagnosis were 181(95%CI 120.2, 241.8) months and 38.5 (95%CI 30.9, 46.1) months. Patient with other variants had estimated mean survival time of 102 (95%CI 73.1, 132.1) months after symptom onset and 43.9 (95%CI 31.3, 56.4) months at diagnosis. Cumulative survival rate at 150 months after symptom onset was 66.7% in Ala97Ser and 0% in other variants. The difference in survival curve for Ala97Ser and other variants at symptom onset was not significant [Breslow (Generalized Wilcoxon), χ2 = 2.92, *p* = 0.087] (Figure 3A). No significant difference was found in survival curve between patient carrying Ala97Ser and other variants at confirmed diagnosis. [Breslow (Generalized Wilcoxon), χ2 = 0.859, *p* = 0.35] (Figure 3B).

Patient at an early disease stage (NAC stage I) tended to have a lower mortality rate, with a median survival time of 48 months (95%CI 39–63 month) and a cumulative survival rate at 60 months of 46.7% [Breslow (Generalized Wilcoxon), χ2 = 4.693, *p* = 0.03]. In contrast, the median survival time of patients at a later disease stage (NAC II, III) was 33 months (95%CI 13.7–55.3), with a cumulative survival rate at 60 months of 0% (Figure 3C) All deaths were observed in patients with NYHA III-IV heart failure.

## Discussion

Male predominance (93%) was observed in this cohort, which was higher than previous reports on ATTRv-CM whose samples were approximately 70% (11–13). This may be due to the small sample size of this study. The underlying mechanism for male predominance is unclear. The small sample size of women did not permit summarization of the sex-related clinical characteristics of ATTRv-CM. However, a recent systematic literature review on sex-related differences in transthyretin amyloid cardiomyopathy revealed that women tended to have lower interventricular septal and posterior wall thicknesses, smaller left ventricular end diastolic diameters and a higher LV ejection fraction. It was postulated that the disparity in the incidence of ATTRv-CM between sexes may be due to either the cardioprotective effects of estrogen or sex-related differences in clinical presentation or disease characteristics (11).

A mixed phenotype was most common presentation in this cohort (82.8%), while a predominantly cardiac phenotype and neurological phenotype was seen in 6.9% of patients each. ATTRv phenotype is associated with *TTR* mutations. While some mutations are associated with a predominantly cardiac phenotype, others are primarily related to a neurological phenotype. Previously reported common cardiac mutations (Val122Ile, leu111Met, Thr60Alr, Ile68Leu) in ATTRv were not identified in our cohort (14). However, we found that non-cardiac mutations such as Asp18Asn, Ser23Asn, Glu42Gly, Gly47Glu, Thr59Lys, Glu61Lys, His88Arg and Ala97Ser were associated with a cardiac phenotype with or without neurologic symptoms. These differences in genotype-phenotype relationship may be the result of their diagnosis at different disease stages.

Ten different *TTR* gene mutations, including 9 missense variants and 1 small deletion mutation, were identified. All of the missense variants have been listed as pathogenic or likely pathogenic in the TTR mutation and ClinVar databases (Table 4) (15–23). The missense mutations were related to the mixed phenotype. Glu61Lys, which was previously found to cause ATTR neuropathy, had never been reported in ATTR-CM. The cardiac mutations Asp18Asn and His68Arg also caused neuropathy in our study. The small deletion *TTR* mutation (c.624_632delGACTTCTCC) was located outside the coding region in exon 3 of the *TTR* gene. As the patient's family history could not be collected due to the limited medical records of the deceased parents, it could not be excluded that this variant of unknown significance occurred by chance in a patient with wild-type ATTR.

**Table 4:** Bioinformatic results of the identified missense *TTR* mutations.

**Sequence variant (mRNA)**	**Mutation (protein variant incl.20–aa signal peptide)**	**Location**	**ClinVar**	**Reported phenotype**	**Reported ethnic group**	**References**
c.112G>A	p.Asp38Asn (Asp18Asn)	Exon 2	Likely pathogenic	H	American	Connors et al. (15) Amyloid 10, 160
c.128G>A	p.Ser43Asn (Ser23Asn)	Exon 2	Likely pathogenic	E, H, PN	Portuguese, American	Connors et al. (16) Amyloid 6, 114
c.185A>G	p.Glu62Gly (Glu42Gly)	Exon 2	pathogenic	SN, H, PN	Japanese, Russian, American	Ueno et al. (17) Biochem Biophys Res Commun 169, 1117
c.200G>A	p.GLy67Glu (Gly47Glu)	Exon 2	pathogenic	H, K, PN	German, Italian	Pelo et al. (18) Amyloid 9, 35
c.224T>G	p.Leu75Arg (Leu55Arg)	Exon 3	pathogenic	LM, PN, E	Chinese, German	Long et al. (19) Ophthalmic Genet 33(1):28–33 Connors (2003) Amyloid 10, 160
c.236C>A	p.Thr79Lys (Thr59Lys)	Exon 3	Likely pathogenic	SN, H, PN	Italian, American (Asian)	Saraiva et al. (20) Hum Mutat 5, 191
c.241G>A	p.Glu81Lys (Glu61Lys)	Exon 3	Pathogenic	PN	Japanese	Shiomi et al. (21) Biochem Biophys Res Commun 194, 1090
c.323A>G	p.His108Arg (His88Arg)	Exon 3	Pathogenic	H	Swedish	Holmgren et al. (22) Amyloid 12, 184
c.349G>T	p.Ala117Ser (Ala97Ser)	Exon 4	Pathogenic	PN, H, E, SN	Chinese, Taiwanese	Tachibana et al. (23) Amyloid 6, 282

*H, heart; PN, peripheral neuropathy; SN, Somatic neuropathy; E, eye; K, kidney; LM, lumber stenosis*.

In this study, 10 different mutations (*n* = 40) have been identified in probands and family members, with the most common being Ala97Ser (*n* = 16; 40%). In addition, Ala97Ser is the most common mutation in symptomatic patient (*n* = 11;37.9%). This was different in the United States, where Val1122Ile (p.Val142Ile) and Thr60Ala (p.T80A) were the most common mutations (14). In European countries, Val30Met was the most frequent mutation in subjects with ATTR in the THAOS registry, followed by Ile68Leu (12). This discrepancy may reflect the heterogeneity of ATTR genotypes between different countries. Ala97Ser has been previously found to be a common ATTR mutation in cohorts of Chinese patients with predominant neurologic phenotypes, including patients from north China and Taiwan. It has also been reported in Chinese Malaysians (10, 24–27). (Supplementary Table 2) Similar with previous studies, male predominance was observed in Ala97Ser. Consistant with other studies, our study found that all patients with Ala97Ser initially presented with a predominantly neurologic phenotype in the early stages of disease. However, the proportion of patients developing cardiomyopathy as the disease progressed was higher (72.7%) than previous reports. Most Ala97Ser patients exhibited a mixed phenotype at their time of diagnosis in our study, which suggests that diagnosis was delayed until a late disease stage. This may be attributed to the difficulty with diagnosing ATTR in patients with isolated peripheral neuropathy symptoms, especially when reg flag symptoms such as carpal tunnel syndrome and lumber stenosis were rarely presented in this cohort. In this situation, family history and genetic examination were important to the early diagnosis of an ATTR patient with a neurological phenotype. On the other hand, CMR and ^99m^Tc-PYP scintigraphy may help to identify myocardial involvement in patients who does not report symptom of heart failure due to limited physical activity caused by neuropathy.

Survival is poor in patients with ATTR cardiac amyloidosis. Although survival varies by genotype, phenotype, and disease stage, most series have reported a median survival of 2.5–3.5 years for patients with heart failure (8). There was a poorer prognosis in the present work for subjects with more severe heart failure, with a 5 year survival rate of <20% for those with NYHA III-IV disease. Several studies have noted the prognostic role of NT-proBNP (28). The NAC staging system includes NT-proBNP and eGFR and has a good prognostic accuracy (29). Patients with advanced NAC stage (stage 2, 3) had a poor prognosis (median survival: 34.5 months, 3-year survival rate: 43%). More advanced NYHA classification at diagnosis also tended to predict a poor prognosis, although there was no statistical significance due to the limited sample size of our study. Phenotype, genotype, initial manifestation and severity of peripheral neuropathy as shown by PND score were not predictive in our study.

The natural average life expectancy is 9 to 13 years after the symptom onset and death usually results from cardiac involvement and cachexia (30). As disease modifying therapy, liver transplantation has been demonstrated to prolong survival. However, the statistics are mostly related to patients with ATTR V30M (31), which was not identified in our study cohort. Different from previous two cohort studies in China, no patient received liver transplantation in our cohort because the following reasons 1) For patients with advanced heart failure, liver transplantation does not provide a complete cure for the disease due to the newly formed amyloid around the preexisting amyloid seeds (32). Combining liver and heart transplantation may be a better strategy. 2) Liver transplantation is restricted by the availability of transplant organs as well as the advanced age and comorbidities of ATTR-CM patients. 3) The need for life-long immunosuppression therapy.

Tafamidis, a small molecule that inhibits the dissociation of transthyretin tetramers, was granted marketing authorization by China Food and Drug Administration for the treatment of inherited or wild type cardiac transthyretin amyloidosis in adult patients in 2020. However, only a few patients could afford this disease modifying therapy because the price of tafamidis was expensive (64,100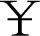/month in 2020, then reduced to 24,650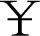/month in 2021). Up to November 2021, only 9.5% (4) patients received tafamidis in our cohort. Fortunately, more patients will have chance to receive this disease modifying therapy since National Health Insurance Administration has issued a national coverage decision to incorporate Tafamidis into medical insurance payouts in 2022.

There are several limitations to our study. First, this was a retrospective study that included a limited sample size. Second, all probands were admitted to our cardiology department first, which results in selection bias that may not permit the description of the actual phenotype-genotype relationship. Third, nuclear scintigraphy was only used in 9 (31%) patients. Expanding the sample size and long-term follow-up of the proband patients and their genotype positive phenotype negative pedigree is needed to understand the relationship between ATTRv genotype and disease course.

## Conclusion

Hereditary ATTRv-CM has heterogeneous phenotypes and genotypes. Most patients had a mixed phenotype that included both cardiomyopathy and neuropathy. Ala97Ser, the most common mutation in South China, initially presented as peripheral and automatic neuropathy and progressed to heart failure at a later disease stage. The prognosis of ATTR was poor, and patients with NYHA III-IV and NAC stage 3 had the worst prognosis.

## Data Availability Statement

The original contributions presented in the study are included in the article/Supplementary Materials, further inquiries can be directed to the corresponding author.

## Ethics Statement

The studies involving human participants were reviewed and approved by the Ethics Committee of Second Xiangya Hospital. The patients/participants provided their written informed consent to participate in this study.

## Author Contributions

SW wrote the protocol, evaluated cardiac involvement, analyzed the results and wrote the manuscript. WP and MP collected the data. LM did the TTR sequencing and analyzed the gene data. DP conceived of the study. BY revised the manuscript and provided guidance for the study. SW, BY, DP, SW, DH, YY, and JH evaluated the patients and made the clinical diagnosis for each patient. All authors contributed to the article and approved the submitted version.

## Funding

This work was supported by grants from the National Nature Science Foundation Youth Project (81600359) and the National Nature Science Foundation General Project (81870336).

## Conflict of Interest

The authors declare that the research was conducted in the absence of any commercial or financial relationships that could be construed as a potential conflict of interest.

## Publisher's Note

All claims expressed in this article are solely those of the authors and do not necessarily represent those of their affiliated organizations, or those of the publisher, the editors and the reviewers. Any product that may be evaluated in this article, or claim that may be made by its manufacturer, is not guaranteed or endorsed by the publisher.
