# Supplementary material for: Clinical Profile and Prognosis of Hereditary Transthyretin Amyloid Cardiomyopathy: A Single-Center Study in South China
Source: Front Cardiovasc Med. 2022 Jun 27;9:900313. doi: 10.3389/fcvm.2022.900313 (PMC9271707; doi:10.3389/fcvm.2022.900313)
Supplement: Supplementary file 1 [file Table_1.DOCX]

Supplement Table 1. The datasets for the nucleotide sequences of *TTR* mutations can be found in GenBank.

| *TTR* mutation | | GenBank accession numbers |
| --- | --- | --- |
| Sequence Variant  (mRNA) | Mutation (Protein Variant incl.20-aa signal peptide) |  |
| c.112G>A | p.Asp38Asn (Asp18Asn) | BankIt2552734 Seq_c.112 OM904529 |
| c.128G>A | p.Ser43Asn (Ser23Asn) | BankIt2552734 Seq_c.128 OM904530 |
| c.185A>G | p.Glu62Gly (Glu42Gly) | BankIt2552734 Seq_c.185 OM904531 |
| c.200G>A | p.GLy67Glu (Gly47Glu) | BankIt2552734 Seq_c.200 OM904532 |
| c.224T>G | p.Leu75Arg (Leu55Arg) | BankIt2552734 Seq_c.224 OM904533 |
| c.236C>A | p.Thr79Lys (Thr59Lys) | BankIt2552734 Seq_c.236 OM904534 |
| c.241G>A | p.Glu81Lys (Glu61Lys) | BankIt2552734 Seq_c.241 OM904535 |
| c.323A>G | p.His108Arg (His88Arg) | BankIt2552734 Seq_c.323 OM904536 |
| c.349G>T | p.Ala117Ser (Ala97Ser) | BankIt2552734 Seq_c.349 OM904537 |
| Exon3 3’UTR c.624_632delGACTTCTCC |  | BankIt2552734 Seq_3*UTR OM904538 |
