# Supplementary material for: Clinical Profile and Prognosis of Hereditary Transthyretin Amyloid Cardiomyopathy: A Single-Center Study in South China
Source: Front Cardiovasc Med. 2022 Jun 27;9:900313. doi: 10.3389/fcvm.2022.900313 (PMC9271707; doi:10.3389/fcvm.2022.900313)
Supplement: Supplementary file 2 [file Table_2.DOCX]

**Supplement table 2. Summary of Clinical manifestation of patients with ATTR Ala97Ser mutation.**

|  | **Number of patients** | **Male (%)** | **Symptom onset age (years, mean±SD，median，range)** | **Progressive polyneuropathy** | **Ventricular wall thickening on echocardiography** | **Heart failure** |
| --- | --- | --- | --- | --- | --- | --- |
| This study | 11 | 10 (90.9%) | 63.3±4.2, 65，53-66 | 11/11 (100%) | 11/11 (100%) | 8/11 (72.7%) |
| Meng et al., 2021 | 7 | 7(100%) | 58.6±4.2 | 7/7 (100%) | 3/6 (50%) | 0 (0%) |
| Scl et al., 2019 | 11 | 6 (55%) | 57, 41-66 | 11/11(100%) | 7/11 (63.6%) | NA |
| Hsu et al., 2017 | 8 | 5 (62.5%) | 61.7±5.5，60, 55-69 | 7/7 (100%) | 7/7 (100%) | NA |
| Chao et al., 2015 | 28 | 25(89%) | 59.9±6.0, 59,48-71 | 28/28 (100%) | NA | NA |
| Liu et al., 2008 | 5 | 3(60 %) | 50.4±5.6, 49,46-60 | 5/5 (100%) | NA | 2 (40%) |
